# Novel Azoles as Antiparasitic Remedies against Brain-Eating Amoebae

**DOI:** 10.3390/antibiotics9040188

**Published:** 2020-04-17

**Authors:** Ayaz Anwar, Mohammad Ridwane Mungroo, Simal Khan, Itrat Fatima, Rafaila Rafique, Kanwal, Khalid Mohammed Khan, Ruqaiyyah Siddiqui, Naveed Ahmed Khan

**Affiliations:** 1 Department of Biological Sciences, School of Science and Technology, Sunway University, Petaling Jaya, Selangor 47500, Malaysia; ayazanwarkk@yahoo.com (A.A.); 15048770@imail.sunway.edu.my (M.R.M.); 2 Department of Biotechnology, Government College University, Lahore 54000, Pakistan; khansimal520@gmail.com or; 3 H. E. J. Research Institute of Chemistry, International Center for Chemical and Biological Sciences, University of Karachi, Karachi 75270, Pakistan; itrat@hotmail.com (I.F.); rafique@gmail.com (R.R.); khalid.khan@iccs.edu (K.M.K.); 4 Institute of Marine Biotechnology, Universiti Malaysia Terengganu, Kuala Terengganu, Terengganu 21030, Malaysia; kanwal.miyanji@gmail.com; 5 Department of Clinical Pharmacy, Institute for Research and Medical Consultations (IRMC), Imam Abdulrahman Bin Faisal University, P.O. Box 1982, Dammam 31441, Saudi Arabia; 6 Department of Biology, Chemistry and Environmental Sciences, American University of Sharjah, Sharjah 26666, UAE; rsiddiqui@aus.edu

**Keywords:** brain-eating amoeba, *Naegleria*, *Balamuthia*, azole, synthesis, nanoparticles

## Abstract

*Balamuthia mandrillaris* and *Naegleria* *fowleri* are opportunistic protozoan pathogens capable of producing infection of the central nervous system with more than 95% mortality rate. Previously, we have synthesized several compounds with antiamoebic properties; however, synthesis of compounds that are analogues of clinically used drugs is a highly desirable approach that can lead to effective drug development against these devastating infections. In this regard, compounds belonging to the azole class possess wide range of antimicrobial properties and used clinically. In this study, six novel benzimidazole, indazole, and tetrazole derivatives were synthesized and tested against brain-eating amoebae. These compounds were tested for their amoebicidal and static properties against *N. fowleri* and *B. mandrillaris*. Furthermore, the compounds were conjugated with silver nanoparticles and characterized. The synthetic heterocyclic compounds showed up to 72% and 65% amoebicidal activities against *N. fowleri* and *B. mandrillaris* respectively, while expressing up to 75% and 70% amoebistatic activities, respectively. Following conjugation with silver nanoparticles, amoebicidal activities of the drugs increased by up to 46 and 36% versus *B. mandrillaris* and *N. fowleri.* Minimal effects were observed when the compounds were evaluated against human cells using cytotoxicity assays. In summary, azole compounds exhibited potent activity against *N. fowleri* and *B. mandrillaris.* Moreover, conjugation of the azole compounds with silver nanoparticles further augmented the capabilities of the compounds against amoebae.

## 1. Introduction

*Balamuthia mandrillaris* and *Naegleria fowleri* are free-living amoebae known to instigate granulomatous amoebic encephalitis (GAE) and primary amoebic meningoencephalitis (PAM), respectively, which are infections of the central nervous system (CNS) [1,2]. Although considered rare conditions, the number of narrated cases of amoebic infection are on the rise. Cases of brain-eating amoebae are usually fatal, comprising a mortality rate of over 95% for *B. mandrillaris* and *N. fowleri* [3,4].

Despite the use of remedies such as miconazole, rifampin, amphotericin B, ornidazole, chloramphenicol, trifluoperazine, dexamethasone, miltefosine, flucytosine, 5-fluorocytosine, metronidazole, ceftriaxone, azithromycin, artesunate, ketoconazole, sulfadiazine, fluconazole, clarithromycin, thioridazine, pentamidine, and itraconazole, in the treatment against the amoebae, the mortality rates remain distressingly high, demonstrating lack of effective treatments and hence, the critical need for the improvement of novel treatments for use against amoebae [5].

Benzimidazoles are heterocyclic compounds that exhibit a wide range of biological properties [6]. Chloro-, bromo- and methyl- derivatives of benzimidazoles were tested against *Acanthamoeba castellanii* [7]. It was reported that benzimidazoles exhibits activity against the amoebae at concentrations as low as 3.5 μmol/L, showing more efficacy than chlorohexidine [7]. It was also reported that the low molecular weight of benzimidazoles facilitates their penetration through membrane, where they inhibit protein kinase and helicase [7]. Benzimidazole also showed effect against other protozoans, such as *Trichinella spiralis*. *Giardia intestinalis*, *Giardia lamblia*, *Entamoeba histolytica*, *Trichomonas vaginalis*, *Leishmania Mexicana* are some protozoans against which benzimidazoles were active with IC_50_ at concentrations as low as 1 μM [8,9,10,11,12]. Benzimidazoles exhibited potent activity against *Trypanosoma brucei rhodesiense* and *Plasmodium falciparum* at concentrations of 30 and 2.1 nM, respectively [13].

Indazoles are heterocyclic compounds processing a broad range of pharmaceutical and biological properties. The derivatives of indazoles exhibits anticancer, antibacterial, anti-inflammatory, antiviral, antioxidants, antidiabetic, antituberculosis, antispermetogenic, and antiproliferative activities [14]. It has been shown that indazoles possess anti-protozoan activity against *Trichomonas vaginalis* and *Trypanosoma cruzi* [15]. Activity of indazoles against *Aspergillus niger*, a fungus, has also been reported [16]. Furthermore, it has been described that indazoles display antimicrobial activity against *Staphylococcus aureus*, *Bacillus megaterium*, *Proteus vulgaris*, *Bacillus substilis,* and *Escherichia coli* [16,17].

Tetrazoles possess anticancer, antifungal, anti-inflammatory, analgesic, anti-hyperlipidemic, antitubercular, and antidiabetic capabilities [18]. Tetrazoles have also shown activity against various fungus including *Candida spp., Cryptococcus neoformans* and *Aspergillus spp.* [19,20,21]. Antiviral activities of tetrazoles have also been reported against HIV-I retrovirus and tobacco mosaic virus [22]. It has also been reported that tetrazoles possess antibacterial activities against *Pseudomonas aeruginosa, Bacillus pumilis, Bacillus subtilis, Escherichia coli,* and *Staphylococcus aureus* [22,23].

Nanoparticle-based materials have shown positive activities against free-living amoebae. In support, we and others have shown recently that amphotericin B, nystatin, fluconazole, cinnamic acid and tannic acid coated with nanoparticles proved to be effective against free-living amoebae [24,25,26].

Considering that benzimidazole, indazole, and tetrazole derivatives display an extensive range of biological activities, we synthesized and characterized novel azole compounds (Table 1) and tested them against *N. fowleri* and *B. mandrillaris*. The azole compounds were tested for their amoebicidal and amoebistatic properties. Moreover, the azoles were also conjugated with silver nanoparticles and characterized, in an attempt to further increase their activity. Azole compounds exhibited potent antiamoebic activity against *N. fowleri* and *B. mandrillaris* and conjugation of the azole compounds with silver nanoparticles further augmented capabilities of the compounds against these amoebae.

## 2. Results

### 2.1. Characterization of Azoles

#### 2.1.1. 2-[3-Bromo-4,5-*bis*(methoxy)phenyl]-1*H*-benzimidazole (A1)

Yield:69%; m.p. 228–230 °C; ^1^H-NMR: (300 MHz, DMSO-*d_6_*): *δ_H_* 12.94 (s, 1H, NH), 7.99 (d, 1H, *J*_2ʹ,6ʹ_ = 3.0 Hz, H-2ʹ), 7.86 (d, 1H, *J*_6ʹ,2ʹ_ = 3.0 Hz, H-6ʹ), 7.66 (d, 1H, *J*_4,5_ = 6.0 Hz, H-4), 7.54 (d, 1H, *J*_7,6_ = 6.0 Hz, H-7), 7.21 (m, 2H, H-5, H-6), 3.96 (s, 3H, 4ʹ-OCH_3_), 3.80 (s, 3H, 5ʹ-OCH_3_); EI-MS: *m/z* (% rel. abund.), 334 [M^+^ + 2] (100), 332 [M]^+^ (91), 319 (48), 254 (9), 238 (31).

#### 2.1.2. 2-(5-Methylfuran-2-yl)-1*H*-benzimidazole (A2)

Yield: 78%; m.p. 241–243 °C; ^1^H-NMR: (300 MHz, DMSO-*d_6_*): *δ_H_* 12.77 (s, 1H, NH), 7.59 (m, 1H, H-4) 7.46 (m, 1H, H-7), 7.20 (m, 2H, H-5, H-6), 7.06 (d, 1H, *J*_5ʹ,4ʹ_ = 3.0 Hz, H-5ʹ), 6.34 (d, *J*_4ʹ,5ʹ_ = 3.0 Hz, 1H, H-4ʹ), 2.40 (s, 3H, 3ʹ-CH_3_); EI-MS: *m/z* (% rel. abund.), 198 [M]^+^ 198.0793; Found 198.0793.

#### 2.1.3. (*E*)-*N*-(2″-Chlorophenyl)-2-(6,6-dimethyl-1-phenyl-1,5,6,7-tetrahydro-4*H*-indazol-4-ylidene)hydrazine-1-carbothioamide (A3) [27]

Yield: 74%, m.p.: 175–177 °C; ^1^H-NMR (400 MHz, DMSO-*d*_6_): *δ*_H_ 10.88 (s, 1H, NH_a_), 10.07 (s, 1H, NH_b_), 8.23 (s, 1H, CH), 7.92 (d, *J_3_*_″_*_4_*_″_ = 7.2 Hz, 1H, H-3″), 7.58 (overlapping multiplet, 5H, H-2′, H-3′, H-4′, H-5′, H-6′), 7.43 (overlapping multiplet, 2H, H-5″, H-6″), 7.29 (t, *J_4_*_″_*_(3_*_″_*_,5_*_″_*_)_* = 6.4 Hz, 1H, H-4″), 2.79 (s, 2H, H-5), 2.65 (s, 2H, H-7), 1.01 (s, 6H, 2CH_3_); ^13^C-NMR (100 MHz, DMSO-*d_6_*): *δ*_C_ 177, 145, 143, 139, 137, 136.5, 130, 129, 128.6, 128.6, 127.3, 127.2, 127.2, 127, 123, 117, 38, 36, 34, 28, 28; FAB (Pos.) MS *m/z* = 424 [M − H]^+1^; HRFAB-MS Calcd for C_22_H_23_N_5_ClS: *m/z* = 424.1363, Found 424.1399.

#### 2.1.4. (*E*)-2-(6,6-Dimethyl-1-phenyl-1,5,6,7-tetrahydro-4*H*-indazol-4-ylidene)hydrazine-1-carbothioamide (A4) [27]

Yield: 72%, m.p.: 234–236 °C; ^1^H-NMR (400 MHz, DMSO-*d*_6_): *δ*_H_ 10.22 (s, 1H, NH), 8.12 (s, 1H, CH), 8.12 (s, 1H, NH_2A_), 7.88 (s, 1H, NH_2B_), 7.56 (m, 4H, H-2ʹ, H-3ʹ, H-5ʹ, H-6ʹ), 7.41 (m, 1H, H-4ʹ), 2.75 (s, 2H, H-5), 2.54 (s, 2H, H-7), 0.97 (s, 6H, 2CH_3_); EI-MS *m/z* (% rel. abund.): 313 (M^+^, 36), 296 (100), 279 (48), 224 (28), 182 (91), 155 (18), 77 (28).

#### 2.1.5. 5-(3’Chlorobenzyl)-1*H*-tetrazole (A5) [28]

Yield: 99%; m.p. 148–150 °C; ^1^H-NMR: (300 MHz, DMSO-*d_6_*): *δ_H_* 16.16 (s, 1H NH), 7.38 (m, 3H, H-4’, H-5’, H-6’), 7.23 (s, 1H, H-2’), 4.31 (s, 2H, CH_2_); EI-MS: *m/z* (% rel. abund.), 196 [M^+^ + 2] (14), 194 [M]^+^ (44), 166 (20), 125 (100); HREI-MS: *m/z* Calcd. for C_8_H_7_ClN_4_ [M]^+^ 194.0359; Found 194.0366; Anal. Calcd. for C_8_H_7_ClN_4_: C, 49.37; H, 3.63; Cl, 18.22; N, 28.79; Found: C, 49.38; H, 3.64; N, 28.77.

#### 2.1.6. 5-Phenyl-1*H*-tetrazole (A6) [28]

Yield: 91%; m.p. 245–246 °C; ^1^H-NMR: (500 MHz, DMSO-*d_6_*): *δ_H_* 16.86 (s, 1H, NH), 8.04 (dd, 2H, *J_2’,3’_* = *J_6’,5’_* = 8.0 Hz, *J_2’,4’_* = *J_6’,4’_* = 2.5 Hz, H-2’, H-6’), 7.60 (m, 3H, H-3’, H-5’, H-4’); ^13^C-NMR (75 MHz, DMSO-d_6_): δ_c_ 131.2 (C-4’), 129.4 (C-2’, C-6’), 127.0 (C-3’, C-5’); IR (KBr, cm^−1^):3128 (N-H), 1609 (C=N); EI-MS: *m/z* (% rel. abund.), 146 [M]^+^ (25), 118 (100), 91 (50), 77 (28); HREI-MS: *m/z* Calcd for C_7_H_6_N_4_ [M]^+^ 146.0592; Found 146.0598; Anal. Calcd. for C_7_H_6_N_4_: C, 57.53; H, 4.14; N, 38.34; Found: C, 57.55; H, 4.16; N, 38.33.

### 2.2. A1, A3, A5 and A6 Showed Significant Amoebicidal Activity against B. mandrillaris

**Figure 1:**
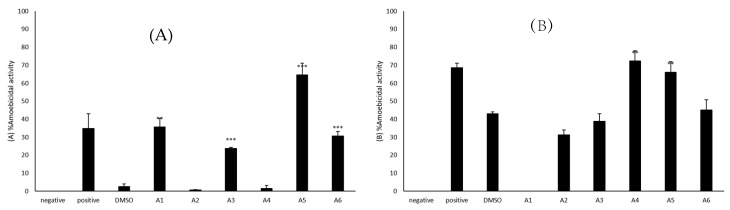
(**A**) The amoebicidal activities of azoles were determined. The results revealed that azoles caused a reduction in the number of viable *B. mandrillaris* cells. (**B**) The results revealed that azoles caused a reduction in the number of viable *N. fowleri* cells. The results are representative of at least three independent experiments performed in duplicates. The results are representative of at least three independent experiments performed in duplicates. The data are presented as the mean ± standard error (***: *p* ˂ 0.001 using 2 sample *t* test; two tailed distribution).

Amoebicidal assays were accomplished to establish the capability of azole compounds to kill the amoebae. The use of 50 *μ*M azole compounds caused a reduction in the number of viable *B. mandrillaris* as compared to the untreated amoebae. A1, A3, A5, and A6 showed activity against *B. mandrillaris* (Figure 1A). A5 caused a percentage cell death of 65% when compared to negative control which was considered as 0% while A1, A6, and A3 caused a percentage cell death of 36, 31, and 24%, respectively. A2 and A4 showed limited amoebicidal activity in comparison to the negative control. However, compared to the solvent control (M = 2.50, SD = 2.57), the compounds A1 (M = 35.62, SD = 6.86), A3 (M = 23.86, SD = 0.81), A5 (M = 64.54, SD = 11.37) and A6 (M = 30.58, SD = 4.68) exhibited significantly higher amoebicidal activities (t(3) = 8.09, *p* = 0.0039; t(4) = 13.75, *p* = 0.0002; t(4) = 9.22, *p* = 0.0008; t(4) = 9.11, *p* = 0.0008).

### 2.3. A4 and A5 Showed Significant Amoebicidal Activity against N. fowleri

Amoebicidal assays were accomplished to establish the capability of azole compounds to kill *N. fowleri*. The use of 50 *μ*M azole compounds exhibited amoebicidal activity against *N. fowleri* as A4 and A5 showed activity against *N. fowleri* compared to the solvent control (Figure 1B). A4 had a percentage cell death of 72% when compared to negative control which was considered as 0% while A5 resulted in 66% amoebicidal activity. As compared to the solvent (M = 43.08, SD = 1.76), the compounds A4 (M = 72.34, SD = 9.95) and A5 (M = 66.19, SD = 9.91) expressed amoebicidal activities that were significantly higher (t(4) = 7.09, *p* = 0.0021; t(4) = 5.6241, *p* = 0.0049). A6, A3, and A2 resulted in a percentage cell death of 45, 39, and 31%, respectively. However, A1 did not cause amoebicidal activity against *N. fowleri*.

### 2.4. All Azoles Showed Significant Amoebistatic Activity against B. mandrillaris

**Figure 2:**
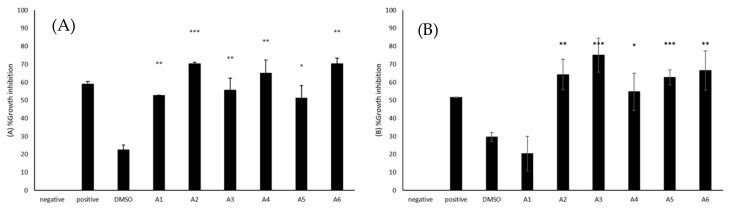
(**A**) The amoebistatic activities of azoles were determined. The results revealed that azoles caused a reduction in the growth of *B. mandrillaris* cells. (**B**) The results revealed that azoles caused a reduction in the growth of *N. fowleri* cells. The results are representative of at least three independent experiments performed in duplicates. The data are presented as the mean ± standard error (*: *p* ˂ 0.05, **: *p* ˂ 0.01 and ***: *p* ˂ 0.001 using 2 sample t test; two tailed distribution).

Amoebistatic assays were accomplished to establish the capability of azole compounds to prevent the growth of the amoebae. The use of 50 μM azole compounds resulted in amoebistatic activities against *B. mandrillaris* (Figure 2A). A2 and A6 exhibited 70% growth inhibition when wells with untreated amoebae were considered as 0%, while A4, A3, A1, and A5 showed 65, 56, 53, and 51% amoebistatic activity, respectively. The amoebistatic activities of the azoles, A1 (M = 52.63, SD = 0.36), A2 (M = 70.33, SD = 1.55), A3 (M = 55.62, SD = 11.43), A4 (M = 64.98, SD = 12.89), A5 (M = 51.24, SD = 11.84), and A6 (M = 70.35, SD = 5.21) were significantly higher than the solvent control (M = 22.58, SD = 4.64), (t(3) = 8.67, *p* = 0.0032; t(3) = 13.42, *p* = 0.0009; t(4) = 4.64, *p* = 0.0097; t(4) = 5.36, *p* = 0.0058; t(3) = 4.01, *p* = 0.0278; t(3) = 10.81, *p* = 0.0017).

### 2.5. Most Azoles Showed Significant Amoebistatic Activity against N. fowleri

Amoebistatic assays were accomplished to establish the capability of azole compounds to inhibit the growth of the amoebae. The use of 50 μM azole compounds resulted in amoebistatic activities against *N. fowleri* (Figure 2B). A3 caused 75% growth inhibition when compared to untreated cells, while the use of A6, A2, A5, A4, and A1 resulted in 67, 64, 63, 55, and 20% amoebistatic activity. A2 (M = 64.31, SD = 12.09), A3 (M = 75.12, SD = 13.31), A4 (M = 54.70, SD = 14.70), A5 (M = 62.74, SD = 5.89), and A6 (M = 66.53, SD = 15.29) showed significantly higher amoebistatic properties when compared with the solvent control (M = 29.61, SD = 3.50), (t(4) = 6.75, *p* = 0.0025; t(4) = 8.10, *p* = 0.0013; t(4) = 4.06, *p* = 0.0153; t(4) = 11.84, *p* = 0.0003; t(4) = 5.76, *p* = 0.0045).

### 2.6. Most Azole Compounds Did Not Exhibit High Cytotoxicity

**Figure 3:**
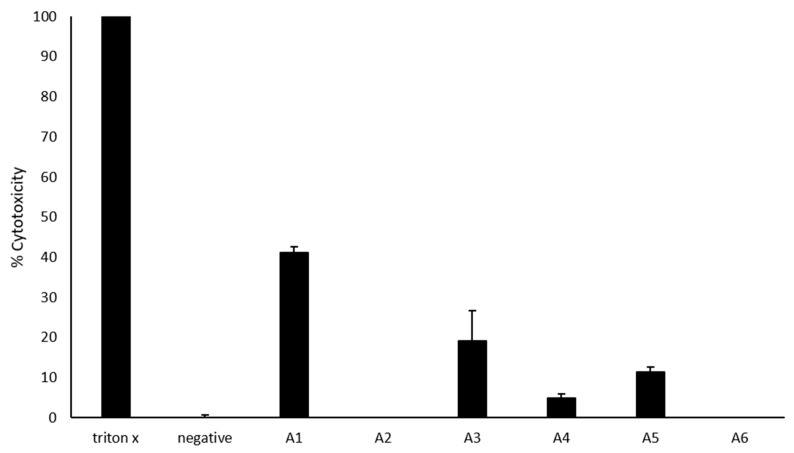
Cytotoxic effects of azoles against host cells. Briefly, 50 *μ*M azoles were incubated with HaCaT cells monolayers for 24 h at 37 °C in a 5% CO_2_ incubator as described in Materials and Methods. The results showed that most azoles have limited host cell damage. The data are presented as the mean ± standard error.

Most of the azoles exhibited low to no cytotoxicity against the human cells (Figure 3). While A2 and A6 exhibited no cytotoxic activities, A4, A5, and A3 exhibited low cytotoxic activities of 5, 11, and 19%, respectively. However, A1 exhibited 41% cytotoxicity.

### 2.7. Characterization of Azoles-AgNPs

**Figure 4:**
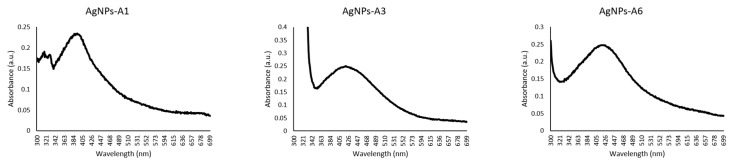
UV-visible spectrum of azoles after conjugation with silver nanoparticles. Peaks can be observed at around 400 nm, suggesting successful conjugation of drugs and nanoparticles.

**Figure 5:**
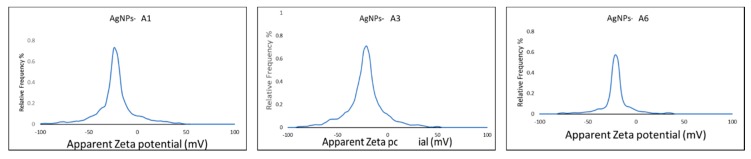
Zeta potential of azoles after conjugation with silver nanoparticles. Peaks can be observed at low millivolts suggesting successful conjugation and formation of small particles.

**Figure 6:**
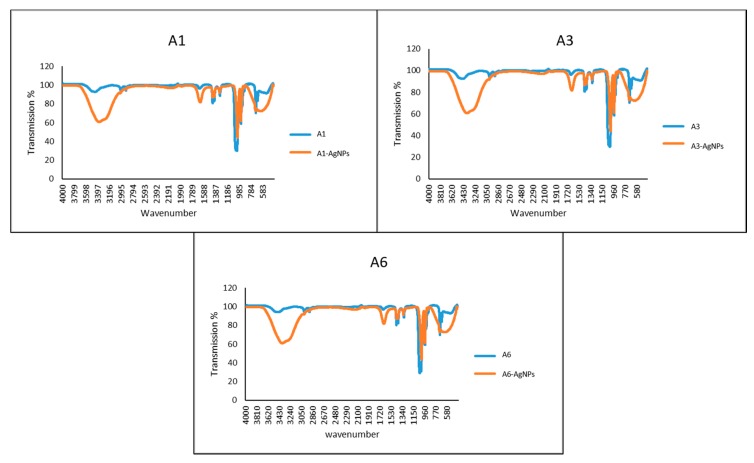
FT-IR spectrum of azoles before and after conjugation with silver nanoparticles. Changes can be seen in the peaks before and after conjugation with silver-nanoparticles, suggesting successful conjugation of drugs and nanoparticles.

The UV-visible spectra of the azole-AgNPs revealed characteristic peaks at around 400 nm, indicating successful formation of azole-AgNPs (Figure 4). The average size for most of the azole-AgNPs was around 100 nm, indicating that small particles were formed. The zeta potential of the azole-AgNPs was found to be −20 mV (Figure 5). Furthermore, differences were observed in the FTIR spectrum of the azole-AgNPs and the azoles before conjugation with AgNPs indication successful formation of azole-AgNPs (Figure 6).

### 2.8. Conjugation with Silver Nanoparticles Significantly Improved Amoebicidal Effects of A4 against B. mandrillaris

**Figure 7:**
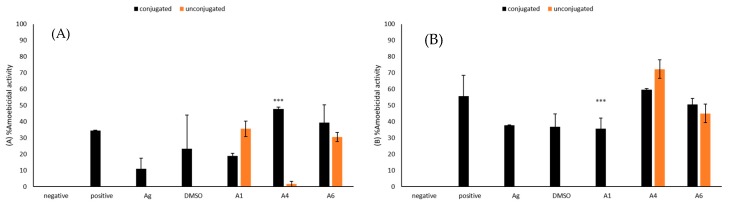
(**A**) The amoebicidal activities of azoles alone and those conjugated with silver nanoparticles were determined. The results show effects of azoles before and after conjugation on *B. mandrillaris* cells. (**B**) The results show effect of azoles before and after conjugation on *N. fowleri* cells. The results are representative of at least three independent experiments performed in duplicates. The data are presented as the mean ± standard error (***: *p* ˂ 0.001 using 2 sample t test; two tailed distribution).

Amoebicidal assays were implemented to establish the ability of conjugation of azole compounds with silver nanoparticles to enhance the amoebicidal activities of the compounds. The activity of A4 increased by 46% following conjugation with silver nanoparticles while that of A6 increased by 9%, respectively (Figure 7A). The amoebicidal activity of A4 after conjugation with silver nanoparticles (M = 47.76, SD = 2.01) was significantly higher when compared to activity of A4 before the conjugation (M = 1.65, SD = 2.85), (t(4) = 25.08, *p* = 0.00002). However, amoebicidal activity of A1 against *B. mandrillaris* was not enhanced through conjugation with silver nanoparticles.

### 2.9. Conjugation with Silver Nanoparticles Significantly Improved Amoebicidal Effects of A1 against N. fowleri

Amoebicidal assays were used to assess ability of conjugation of azole compounds with silver nanoparticles to enhance their amoebicidal activities against *N. fowleri*. Amoebicidal activity of A1 increased by 36% following conjugation of azole compounds with silver nanoparticles while amoebicidal activities of A6 increased by 6% (Figure 7B). The increase in amoebicidal activity of A1 after conjugation with silver nanoparticles (M = 35.72, SD = 9.09) as compared to before conjugation (M = 0, SD = 0) was significant (t(4) = 9.63, *p* = 0.0007). However, conjugation of A4 with silver nanoparticles did not enhance their activity against *N. fowleri*.

## 3. Discussion

The synthesis of compounds that are analogues of clinically used drugs is a highly desirable approach that can lead to effective drug development. Compounds belonging to the azole class possess wide range of antimicrobial properties and are used clinically as antifungal and antiparasitic agents. Considering that benzimidazole, indazole, and tetrazole derivatives exhibit an extensive range of biological activities, we synthesized and characterized novel azole compounds and evaluated them against *N. fowleri* and *B. mandrillaris* for their amoebicidal and amoebistatic properties. Moreover, the azoles were also conjugated with silver nanoparticles to further increase their activity.

Amongst the six tested diverse azoles, A5 (tetrazole) showed significant activity for both amoebicidal and amoebistatic assays against both *N. fowleri* and *B. mandrillaris* while exhibiting limited cytotoxicity towards human cells. A1, A3, and A6 also showed both amoebicidal and amoebistatic activities against *B. mandrillaris*. A4 (indazole) also expressed both amoebicidal and amoebistatic activities against *N. fowleri*. The activity of A4 against *B. mandrillaris* increased when conjugated with silver nanoparticles while that of A1 (benzimidazole) was enhanced against *N. fowleri*.

Azoles are a class of compound which is known to inhibit the synthesis of ergosterol. Several azole compounds, such as ketoconazole, fluconazole, and itraconazole, have already been shown to be effective against brain-eating amoebae [5]. Indazoles have been shown to act by causing the production of free radicals, such as OH radicals as well as inhibition of oxygen uptake [29]. Benzimidazoles have been reported to work by inhibition of the polymerization of tubulin. Small sizes of nanoparticles enhance both pharmacokinetics and pharmacodynamics of drugs [30]. Nanoparticles have shown great promise as efficient carriers which enhance the delivery of drugs that have limitations such as unspecific targeting and poor solubility [31]. It was also shown that the conjugation of azoles with nanoparticle can enhance their activity against brain-eating amoebae [24]. This supports our findings which show that the conjugation of silver nanoparticles with azoles improves their anti-amoebic activities.

However, only amoebicidal and amoebistatic activities of the azoles *in vitro* were investigated while other factors such as the ability of the drugs to pass through the blood-brain barrier was not determined. The effect of the drugs on the ability of the amoebae to encyst and excyst and the precise mode of action these compounds against amoebae was also not part of the current study. 

In conclusion, benzimidazole, indazole, and tetrazole derivatives possess anti-amoebic activities that can be enhanced through conjugation with nanoparticles.

## 4. Methods

### 4.1. Human Cervical Adenocarcinoma Cells (Hela) Cultivation

Human cervical adenocarcinoma cells (HeLa) cells were used as a feeder layer for *B. mandrillaris* and *N. fowleri*. (HeLa cells (ATCC^®^ CCL-2™) were attained from American Type Culture Collection and cultured in Roswell Park Memorial Institute (RPMI) 1640 medium, supplemented with 10% foetal bovine serum (FBS), 1% minimum essential medium amino acids, 1% L-glutamine and 1% antibiotics (supplemented RPMI-1640) at 37 °C and 5% CO_2_. When full and confluent, trypsin was used to detach the cells, collected at 1258× *g* for 5 min and then seeded into plates or flasks [32,33].

### 4.2. Cultivation of Amoebae

*N. fowleri* cells (ATCC 30174) and *B. mandrillaris* cells (ATCC 50209) were acquired from American Type Culture Collection and cultivated in RPMI-1640 media supplemented with 1% antibiotics with HeLa cell monolayers used as a feeder layer at 37 °C and 5% CO_2_ as previously cultured [24,34].

### 4.3. Amoebicidal Assays

Amoebicidal assays were accomplished as formerly explained [24]. Briefly, 5 × 10^5^ amoebae cells were kept for 24 h at 37 °C and 5% CO_2_ with 50 μM azole compounds. While positive controls used were amphotericin B and ketoconazole, RPMI-1640 alone was used as negative control and respective solvents were used as solvent control. Trypan blue exclusion assay, which consists of using 0.1% trypan blue to differentiate between live (unstained) and dead (stained) cells and count the cells using a haemocytometer.

### 4.4. Amoebistatic Assays

5 × 10^5^ amoebae cells were kept for 24 h at 37 °C and 5% CO_2_ with 50 μM azole compounds on HeLa monolayers as feeder cells. While positive controls used were amphotericin B and ketoconazole, RPMI-1640 alone was used as negative control and respective solvents were used as solvent control. Trypan blue exclusion assay which consists of using 0.1% trypan blue to distinguish between live (unstained) and dead (stained) cells and count the cells using a haemocytometer. 

### 4.5. Cytotoxicity Assays

Cytotoxic effects of the drugs were determined as previously described [32,35]. 50 μM azole compounds was incubated on HeLa cells for 24 h at 37 °C and 5% CO_2_ in RPMI-1640 media. The level of lactate dehydrogenase (LDH) enzyme in the RPMI-media which indicates cytotoxic effects of drugs was determined by means of a cytotoxicity detection kit (Roche Applied Science). The percentage cytotoxicity was calculated: [(Absorbance of sample - Absorbance of untreated cells)/(Absorbance for total LDH release - Absorbance of untreated cells)] × 100 = percentage cytotoxicity.

### 4.6. Synthesis of Azoles

#### 4.6.1. Overall Technique for the Synthesis of Benzimidazoles (A1 and A2)

Benzimidazole derivatives were synthesized by protocol described previously [36]. Briefly, *o*-phenylene diamine derivative (1 mmol) and substituted benzaldehyde (1 mmol) were taken in *N,N*-dimethylformamide (10 mL) into a 100 mL round-bottomed flask. The catalytic amount of sodium metabisulfite (Na_2_S_2_O_5_) was added into it and solution was refluxed for 4 h. Progress of reaction was checked by thin layer chromatography (TLC). After completion of reaction, mixture was poured onto crushed ice (100 mL). Precipitates were appeared immediately which were filtered. The obtained solid crude products were crystallized from ethanol. Compounds were structurally characterized by various spectroscopic techniques.

#### 4.6.2. Overall Technique for the Synthesis of (*E*)-2-(6,6-dimethyl-1-phenyl-1,5,6,7-tetrahydro-4*H*-indazol-4-ylidene)hydrazine-1-carbothioamide (A4)

In a 50 mL round-bottomed flask dimedone (1 mmol), dimethylformaide dimethylacetal (DMF-DMA) (1 mmol), and phenylhydrazine (1 mmol) were added in dry ethanol (10 mL) and refluxed at 70 °C for 3 h. Then thiosemicarbazide (1 mmol) was added and refluxed for further 2 h. Reaction was monitored by TLC. Precipitation indicated the end point which were collected by filtration, washed with hexane and were purified through crystallization from ethanol [27].

#### 4.6.3. Overall Technique for the Synthesis of Tetrazoles (A5 and A6)

In an archetypal procedure, 5-aryl-1*H*-tetrazoles were synthesized by adding aryl nitriles (1 eq.), sodium azide (1.2 eq.), and ammonium chloride (1 eq.) in DMF, the mixture was refluxed for 24 h. Progress of the reaction was monitored by thin layer chromatography. After completion of the reaction, 2.5 mL of 2 M NaOH was added and the solution was stirred for half an hour. The reaction mixture was concentrated on reduced pressure and dissolved in water. 3 M HCl was added to the reaction mixture dropwise until precipitates formed. The precipitates were filtered and washed with distilled water. The title compounds were obtained in moderate to high yields [28].

### 4.7. Nanoparticle Conjugation

Drugs were conjugated with silver nanoparticles (AgNPs) as previously described [37]. Azole compounds were magnetically stirred with silver nitrate, followed by the addition of reducing agent, sodium borohydride. The formation of yellow-brown solution would indicate the formation of drug-AgNPs nano-conjugates. Varying ratios of drug to AgNPs was used and the solutions were further stirred for 1 h to optimize yield.

### 4.8. Characterization of Nanoparticle Conjugation

#### 4.8.1. Spectrometric Analysis 

The absorbance of the azoles-AgNPs were measured in the UV-visible spectrum to detect the characteristic surface plasmon resonance band of drugs-AgNPs as described previously [37]. In brief, 1 mL of azoles-AgNPs was placed into a plastic cuvette and the absorbance of the solution were measured at wavelengths ranging from 190 to 800 nm. Reduction of silver ions to silver nanoparticles usually results in the formation of characteristic peak around 400 nm.

#### 4.8.2. Dynamic Light Scattering

Dynamic light scattering was performed at 25 °C and 90 °C inclination to analyse the distribution of the sizes of the azoles-AgNPs as previously described [25]. A particle size analyzer (Litesizer 500) was used to analyse the azoles-AgNPs placed in a plastic cuvette to generate a size distribution. The same apparatus was used to analyse the zeta potential of the azoles-AgNPs placed in dip cells.

#### 4.8.3. Fourier-Transform Infrared Spectroscopy

Fourier-transform infrared spectroscopy was used to determine and compare the absorbance of azole and azole-AgNPs in the range of 400–4000 cm^−1^ as previously described [25]. The spectrum produced contains characteristic peaks of different functional groups and bonds.

## Figures and Tables

**Table 1:** The table shows the structures and IUPAC names of the azoles used.

Class of Compound	Code	Structure	IUPAC Name
Benzimidazole	A1	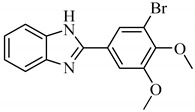	2-[3-Bromo-4,5-*bis*(methoxy)phenyl]-1*H*-benzimidazole
Benzimidazole	A2	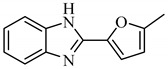	2-(5-Methylfuran-2-yl)-1*H*-benz]imidazole
Indazole	A3	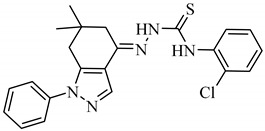	(*E*)-*N*-(2-Chlorophenyl)-2-(6,6-dimethyl-1-phenyl-1,5,6,7-tetrahydro-4*H*-indazol-4-4ylidene)hydrazine-1-carbothioamide
Indazole	A4	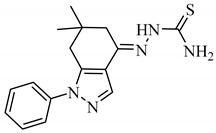	(*E*)-2-(6,6-Dimethyl-1-phenyl-1,5,6,7-tetrahydro-4*H*-indazol-4-4ylidene)hydrazine-1-carbothioamide
Tetrazole	A5	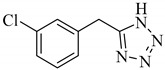	5-(3-Chlorobenzyl)-1*H*-tetrazole
Tetrazole	A6	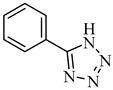	5-Phenyl-1*H*-tetrazole
